# Does dispatcher-assisted bystander CPR improve outcomes from adult out-of-hospital cardiac arrest?

**DOI:** 10.29045/14784726.2019.03.3.4.23

**Published:** 2019-03-01

**Authors:** Ffion Barham, Stephanie Bailey, Blair Graham

**Affiliations:** University Hospitals Plymouth NHS Trust; University Hospitals Plymouth NHS Trust; University Hospitals Plymouth NHS Trust

**Keywords:** cardiopulmonary resuscitation, emergency medical dispatch, emergency medical services

## Abstract

This short review addresses the evidence behind dispatcher-assisted CPR (DA-CPR) and whether it contributes to overall survival of out-of-hospital cardiac arrest (OHCA). Six papers directly addressed the review question and were selected for appraisal, including one systematic review. The outcomes of these studies demonstrate variable results from the implementation of DA-CPR strategies. While DA-CPR has some utility as a substitute for spontaneously delivered bystander CPR, available evidence suggests there is scope to improve. Further work should focus on the identification and adoption of more effective protocols.

## Three-part question

In [adult patients suffering out-of-hospital cardiac arrest (OHCA)] does [emergency medical services dispatcher-assisted CPR (DA-CPR)] improve [clinical outcomes]?

## Clinical scenario

A 65-year-old man is found by passers-by on a riverside path. He is unresponsive and not breathing. The bystanders contact emergency medical dispatch for assistance but do not attempt CPR. The patient has persistent asystole and no return of spontaneous circulation, and is declared dead. Would emergency medical DA-CPR have increased the odds of a favourable outcome?

## Search terms

((basic life support) OR (cardiopulmonary resuscitation) OR CPR OR (out of hospital cardiac arrest)) AND (coaching OR dispatcher) AND ((quality) OR (depth) OR (rate) OR (compression*) OR (ventilation*) OR (Return of Spontaneous Circulation) OR (ROSC) OR (Length of Stay) OR (Mortality) OR (survival) OR (recovery)).

Limits: January 2008–November 2018.

## Search outcomes

Studies examining the administration and/or quality of DA-CPR in relation to clinical outcomes in adults were included. Non-English papers, conference abstracts, letters, case reports or studies conducted exclusively in paediatric populations or simulated scenarios were excluded.

**Figure 1:**
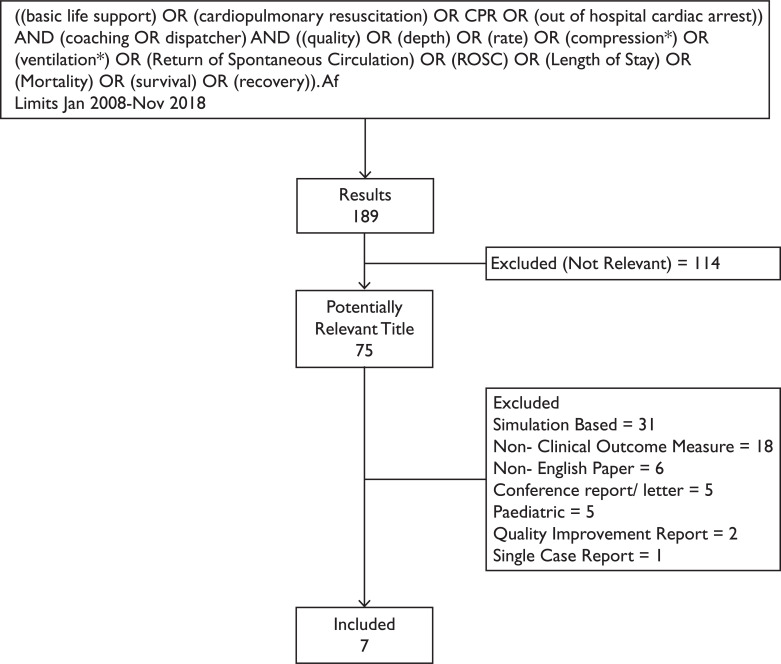
Figure 1. Flow diagram of search outcomes.

Out of 189 results, 75 potentially relevant titles were identified, of which seven papers met the inclusion criteria and were selected for appraisal (Figure 1). This included one systematic review.

## Search results

Search results are presented in Table 1.

**Table 1.** Search outcomes.

Authors, date and country	Study type, methods and outcomes (where stated)	Description of included studies/population	Key results
Bohm, Vaillancourt, Charette, Dunford, & Castrén, 2011, Sweden	Systematic review. Review of papers on bystander CPR with or without dispatcher assistance. Primary outcome: survival to hospital discharge.	Papers published between 1985 and 2009 (n = 5).	Two studies demonstrated trends towards increased survival to discharge with dispatcher coaching (11.4% and 33%, respectively). One study showed that dispatcher coaching increased survival to discharge vs. no bystander CPR at all (OR 1.45) but that spontaneous bystander CPR had the highest survival overall (21.4% vs. 15.1%). Two studies showed no difference.
Park et al., 2018, South Korea	Before and after intervention study. Assessed the impact of a 3-part intervention consisting of (1) improved DA-CPR, (2) co-responder dispatch to OHCA and (3) use of CPR feedback devices. Primary outcomes: return of spontaneous circulation (ROSC), survival to discharge and neurologically favourable survival from OHCA.	Patients with OHCA before (n = 6201) and after (n = 6469) intervention.	The 3-part intervention significantly improved all outcomes including survival to discharge (10.9% vs. 9.6%; p < 0.0001). Multivariate analysis revealed that compared to no bystander CPR, DA-CPR was associated with increased ROSC (OR 1.41, 95% CI 1.20–1.66), survival to discharge (OR 1.14 95% CI 0.97–1.14) and neurologically favourable survival (OR 1.45 95% CI 1.18–1.77).
Song et al., 2014, South Korea	Before and after intervention study. Assessed impact of standardised DA-CPR protocol in one city. Primary outcome: survival to discharge. Secondary outcomes: survival to discharge with a favourable neurological outcome and rates of bystander CPR.	Adult aged 15 or over with out-of-hospital cardiac arrest with presumed cardiac aetiology (n = 8494).	Survival to discharge increased from 7.1% to 9.4% (OR 1.12 95% CI 1.12–1.66) post-intervention. Favourable neurological outcome increased from 2.0% to 3.5% (OR 1.69 95% CI 1.21–2.37).
Harjanto et al., 2016, Singapore	Before and after intervention study. Assessed impact of DA-CPR training programme in Singapore. Primary outcomes: survival to admission, 30-day survival and good functional recovery (Glasgow Pittsburgh Overall Performance Categories 1 or 2).	Adults with cardiogenic OHCA 2010–2013 (n = 2968).	Only spontaneous bystander CPR achieved significantly better improved survival to hospital admission (OR 1.39 (95% CI 1.12–1.74)), 30-day survival (OR 2.07 (95% CI 1.41–3.02)) and good functional recovery (OR 2.70 (95% CI 1.65–4.40)). DA-CPR showed trends towards increased rates of ROSC and 30-day survival, compared to no CPR, but did not reach significance. Those receiving spontaneous bystander CPR had better functional recovery than the no CPR group (OR 2.70 (95% CI 1.65–4.40)).
Ro et al., 2017, South Korea	Prospective observational study using national database. Primary outcome ‘good neurological recovery’ at discharge, defined as cerebral performance category (CPC) 1 or 2. Secondary outcomes were survival to discharge and ROSC.	Adults (18 or older) OHCA of presumed cardiac cause 2012–2014 (n = 37,924).	DA-CPR resulted in increased incidence of CPC 1 or 2 versus no bystander CPR (4.8% vs. 2.1%) but spontaneously delivered bystander CPR was better (5.2%). An identical pattern was observed in relation to survival to discharge (7.3% DA-CPR vs. 8.4% SD-CPR vs. 4.8% no CPR) and ROSC (5.3% vs. 5.9% vs. 2.5%).
Wu et al., 2018, United States	Retrospective observational study. Assessed impact of DA-CPR on survival to hospital discharge (primary outcome) and favourable neurological status (secondary outcome).	Adult (18 or older) OHCA of presumed cardiac aetiology 2011–2014 (n = 2310).	DA-CPR resulted in improved survival (OR 1.51 95% CI 1.04–2.18) and favourable neurological outcome (OR 1.56 95% CI 1.06–2.31) compared to no CPR. There was no difference in measured outcomes between DA-CPR and spontaneously delivered bystander CPR.
Takahashi et al., 2018, Japan	Retrospective observational study. Compared DA-CPR to spontaneous bystander CPR. Outcomes: rate of shockable rhythm on initial ECG, ROSC in the field. Secondary outcomes: CPC 1 or 2 at 1 month.	Analysis of nationwide Utstein Japanese database 2008–2012 (n = 37,889 cases identified).	DA-CPR increased odds of shockable rhythm on arrival (OR 1.75 (95% CI 1.67–1.85)), ROSC (OR 1.42 (95% CI 1.33–1.52)) and neurologically favourable outcome (OR 1.67 (95% CI 1.55–1.80)) compared to no CPR. Spontaneously delivered bystander CPR showed the most favourable outcomes overall.

## Comments

The use of national registries facilitated collection of large amounts of data in all five primary research studies included. However, studies were conducted in a range of international systems where differences in dispatch and emergency medical services (EMS) response protocols may limit external validity. Only one prospective study was identified.

Six out of seven identified studies suggest that DA-CPR may modestly improve the odds of both survival to discharge and favourable neurological outcome from OHCA. Three studies independently report that DA-CPR was inferior to spontaneously delivered bystander CPR. Explanations for the latter may include the presence of trained responders, more rapid initiation of chest compressions and higher quality chest compressions. A single study attempted to assess DA-CPR administration at the time of EMS arrival, and found chest compressions to be of poor quality.

Future work should focus on the comparative effectiveness of DA-CPR protocols in terms of quality of CPR delivery, and effect on clinical outcomes. This may allow for the identification and more widespread adoption of effective protocols or highlight the need for further development in this area. As spontaneously delivered CPR seems superior to DA-CPR, provision of the latter should not be seen as a substitute for widespread public training initiatives.

## Clinical bottom line

Current DA-CPR strategies modestly improve the odds of favourable outcome from OHCA. However, DA-CPR may be inferior to spontaneously delivered bystander CPR. Based on current evidence, opportunities for public training should be maximised and supported by evidence-based DA-CPR protocols.

## Conflict of interest

None declared.

## Funding

None.
